# Disability inclusiveness of government responses to COVID-19 in South America: a framework analysis study

**DOI:** 10.1186/s12939-020-01244-x

**Published:** 2020-08-03

**Authors:** Dikaios Sakellariou, Ana Paula Serrata Malfitano, Elena S. Rotarou

**Affiliations:** 1 School of Healthcare Sciences, Cardiff University, Eastgate House, Newport Road 35-43, Cardiff, CF24 0AB UK; 2 Department of Occupational Therapy, Postgraduate Program in Occupational Therapy, Universidade Federal do São Carlos, São Carlos, SP Brazil; 3 Faculty of Medicine and Science, Universidad San Sebastián, Santiago, Chile

**Keywords:** COVID-19, Convention on the Rights of Persons with Disabilities, Disabled people, Government response, Inclusiveness, South America

## Abstract

**Background:**

Disabled people are particularly exposed to the risks of COVID-19, as well as to the measures taken to address it, and their impact. The aim of the study was to examine the disability-inclusiveness of government responses to COVID-19 in four South American Countries: Argentina, Brazil, Chile, and Peru.

**Methods:**

We conducted documentary research, using framework analysis to analyse reports, legislation, decrees, and other official documents that communicated measures taken in response to the pandemic, published from February 1st until May 22nd, 2020. We included documents reporting measures that affected disabled people either directly (measures specifically designed for disabled people) or indirectly (measures for the general population). We developed an analytical framework based on recommendations for disability-inclusive response to COVID-19 published by the Economic Commission for Latin America and the Carribean, the World Health Organisation, and other international organisations.

**Results:**

We analysed 72 documents. The findings highlight that while some positive measures were taken, the needs of disabled people were not fully considered. Several countries published recommendations for a disability-inclusive response to COVID-19, without ensuring their translation to practice. All countries took at least some steps to ensure access to financial support, health, and education for disabled people, but at the same time they also implemented policies that had a detrimental impact on disabled people. The populations that are most exposed to the impacts of COVID-19, including disabled people living in institutional care, were protected in several cases only by recommendations rather by legislation.

**Conclusions:**

This study illustrates how the official government responses taken by four countries in the region – while positive, in several aspects – do not fully address the needs of disabled people, thus further disadvantaging them. In order to ensure response to COVID − 19 is disability inclusive, it is necessary to translate recommendations to practice, consider disabled people both in mainstream policy and in disability-specific measures, and focus on the long-term reconstruction phase.

## Introduction

During the initial phase of the coronavirus disease 19 (COVID-19) pandemic, the virus was often referred to as the ‘great leveller’ that collapsed differences between people, exposing them to the same risks. This myth was soon debunked: far from being a great leveller, the pandemic has exacerbated inequalities, with protection against the risk of infection, access to treatment, and impacts of public health measures disproportionately affecting the most disadvantaged populations, including the poor, people in precarious employment, people living with chronic conditions, and people belonging to ethnic minorities [1, 2]. Disabled people are particularly exposed to the risks of the pandemic, as well as to the measures taken to address it, and their impact [3–5]. In this article, we examine official government responses to the COVID-19 pandemic in four South American countries – Argentina, Brazil, Chile, and Peru – with the aim of exploring the extent and forms of disability inclusion in government responses to COVID-19. While the existence of a state discourse in the form of legislation, or other form of official government response, does not preclude the existence of disability-based discrimination, it affords disabled people legal recognition and protection of their rights, and as such, its importance cannot be underestimated.

There are 1 billion disabled people globally [6], including over a third of people over 60, the age group with the highest COVID-19 mortality. Disabled people face the effects of structural disadvantage, leading to increased barriers to accessing healthcare, despite increased health needs [7, 8], and increased rates of poverty, lower literacy levels, lower insurance coverage rates, and lower employment compared with the general population [6]. Disabled people may be exposed to an increased risk of contracting the virus because they cannot always maintain the physical distancing measures, especially if they require personal assistance. They are at particular risk from responses to COVID-19, due to interrupted social support and lack of accessible communication, among other factors [1]. They are also more likely to become severely ill due to COVID-19, since many disabled people live with other underlying conditions, such as heart problems, diabetes, and respiratory illness. Nevertheless, there are widespread concerns that disabled people may be less likely to receive needed life-saving treatment and that they may be disproportionately affected by the measures taken by several governments to control the pandemic [3, 4].

On May 22, 2020, the World Health Organisation (WHO) announced that the epicentre of the pandemic had moved to South America. COVID-19 cases have now been detected in all countries in South America [9], with Brazil and Peru being particularly affected [10], putting already overstretched healthcare systems under particular strain [11–13] (see Table 1). More than 70 million disabled people live in Latin America and the Caribbean region, and they are among the most excluded parts of the population, with high levels of poverty and unemployment [14]. The Economic Commission for Latin America and the Caribbean (ECLAC) highlights disabled people as being at increased risk of being impacted by the pandemic, and by the measures taken to address it [15].

**Table 1:** Demographic, socioeconomic, health-related, and COVID-19-related characteristics of Argentina, Brazil, Chile, and Peru

	Argentina	Brazil	Chile	Peru
Population^a^	44,494,502	209,469,333	18,729,160	31,989,256
GDP per capita (USD)^a^	11,684	9001	15,923	6941
GDP per capita (PPP, USD)^a^	23,300	14,952	24,763	13,094
Population living in poverty (%)^b^	24.4	19.4	8.6	16.8
Population living in extreme poverty (%)^b^	3.6	5.4	2.3	3.7
Gini index^a^	0.414	0.539	0.488	0.433
Disabled people (% of population)^c^	12.9	23.9	16.8	5.2
Physicians (per 10,000 people)^d^	39.6	21.5	10.8	12.7
Nurses and midwifes (per 10,000 people)^d^	26	97	9	14
Hospital beds (per 10,000 people)^d^	50	22	22	16
Current health expenditure (% of GDP)^e^	7.5	11.8	8.9	5.1
Confirmed cases^f^	153,520	2,287,475	341,304	375,961
Total COVID tests per 1000 people^g^	11.15	11.93	76.62	10.45
Reported deaths^f^	2847	85,238	8914	17,843
Total confirmed number of deaths per million people^g^	60.23	401.01	466.31	541.16

Sources

^a^World Bank https://data.worldbank.org

^b^Economic Commission for Latin America and the Caribbean https://estadisticas.cepal.org/cepalstat/perfilesNacionales.html?idioma=english

^c^Argentina, Chile, Peru: United Nations Disability Statistics https://unstats.un.org/unsd/demographic-social/sconcerns/disability/statistics/#/countries, Brazil: Brazil Census https://agenciadenoticias.ibge.gov.br/en/agencia-news/2184-news-agency/news/17044-pessoas-com-deficiencia-adaptando-espacos-e-atitudes-3

^d^United Nations Development Program http://hdr.undp.org/sites/default/files/covid-19_and_human_development.pdf

^e^Argentina, Brazil, Peru: United Nations Development Program http://hdr.undp.org/sites/default/files/covid-19_and_human_development.pdf, Chile: Organisation for Economic Development and Cooperation https://data.oecd.org/healthres/health-spending.htm

^f^John Hopkins University https://coronavirus.jhu.edu/map.html

^g^Our World in Data https://ourworldindata.org/coronavirus#coronavirus-country-profiles Figures as of July 25, 2020

Note: All sources last accessed on July 25, 2020

Some governments in the region, and globally, have linked disability to an inherent vulnerability and poorer chances of survival, to justify discrimination in the form of differential access to services. Furthermore, there is a high gap in disability prevalence between income quintiles, with disability being more prevalent among poor people. This gap widens with age, which is when people might lose social protections associated with employment, such as insurance [16]. A large percentage of the population (over 30% in Brazil, and around 20% in Argentina) live in precarious or segregated housing, often in urban slums, such as *favelas* in Brazil or *villas de emergencia* in Argentina [16]. Public health recommendations for physical distancing and hand-washing are often not realistic for slum residents, including disabled people, who may lack personal space and necessities, such as clean water. Overcrowded prisons, where disabled people are overrepresented [4], poorly resourced residential care (often acting as zones of social abandonment [17]), and a large population of indigenous people suffering the effects of discrimination [18], further complicate the situation, as does the ongoing dengue fever outbreak [12], particularly affecting Brazil. All these factors might disadvantage even further people who already have limited ways to protect themselves.

While article 11 of the United Nations Convention on the Rights of Persons with Disabilities (CRPD) stipulates that member states should take “all necessary measures to ensure the protection and safety of persons with disabilities in situations of risk” [19], there have been widespread reports of disabled people facing problems accessing treatment for COVID-19 while many of the public health measures implemented to contain the virus are not taking into account the needs of disabled people. Catalina Devandas, the UN Special Rapporteur on the rights of persons with disabilities, stated that disabled people feel that governmental responses to the COVID-19 pandemic have nor considered their needs, leading to measures that might exacerbate disabled people’s exclusion from society [20].

## Methods

We conducted a documentary approach-based study [21], using framework analysis to examine the official governmental responses to COVID-19 [22]. We focused on four South American countries: Argentina, Brazil, Chile, and Peru, which have a collective population of about 305 million people, accounting for over 72% of the total population of South America. Chile is a high-income country while the other three are upper middle-income countries [23].

### Data sources

We used sources published from February 1st until May 22, 2020, in the form of documents from the agencies coordinating the COVID-19 response and relevant governmental bodies. These documents were treated as raw data sources. We looked at decisions taken by national and federal governments, but not at those by local, provincial, or State governments. We included documents reporting measures that affected disabled people either directly (measures specifically designed for disabled people) or indirectly (measures not designed specifically for disabled people but that have an impact on their life). Information was accessed by reviewing reports, legislation, decrees, and other official documents that communicated measures taken in response to the pandemic. To locate these sources, we searched the websites of the ministries responsible for health, employment, social support, and transportation of the included countries, and also the COVID-19-related government websites for each country. Information was also accessed through the governmental bodies responsible for disability-related matters, and through the government gazettes.

We also included sources in the form of reports and other communications by disabled people’s organisations and reports by the WHO, the United Nations, and ECLAC. Information collated about measures implemented by each country was triangulated with information on country-specific measures collected by ECLAC. No discrepancies or omissions were noted as a result of this process.

We accessed all sources in their original language, which was Portuguese for documents from Brazil, Spanish for sources from all other countries, and English or Spanish for sources from international organisations (see Supplementary material 1).

### Data analysis

We conducted a framework analysis of documents. This method is often used in applied qualitative research with the aim to influence policy. Based on a thematic framework identified from existing literature and informed by the data, framework analysis identifies commonalities and differences in the data, and then focuses on establishing patterns [22]. Analysis involved the following five steps:

1. *Familiarisation, preliminary reading/ research notes:* In this step, we did an initial reading of the data set and also of related literature on disability inclusion.
2. *Identifying a thematic framework:* Ritchie and Spencer describe this step as “*beginning the process of abstraction and conceptualisation”* ([22]:179). We developed a framework based on guidelines for disability inclusion during COVID-19 issued by the following organisations: ECLAC [3], the International Labour Organisation [24], the Ibero-American General Secretariat (Secretaría General Iberoamericana) [25], the United Nations Office of the High Commissioner for Human Rights [4], and the World Health Organisation [26]. Table 2 presents the thematic framework we developed from the synthesis of the different recommendations (see Supplementary material 2 for the full list).
3. *Indexing:* We applied the thematic framework to the entire dataset, looking for information for each of the areas, while carefully considering whether any adjustments needed to be made to the framework, based on the data.
4. *Charting:* In this stage, we extracted information that was relevant and inserted it into a matrix based on the thematic framework. We included both disability inclusion and exclusion-related information.
5. *Mapping:* In the last step, we looked at patterns across the dataset, focusing on the nature and extent of disability inclusion.

**Table 2:** Thematic framework

Area	Explanation
**Accessible information**	Provision of all information in accessible formats, including sign language translation, Braile script, and easy read.
**Access to healthcare**	Removal of financial barriers to care, and measures taken to ensure equitable access to healthcare, including measures addressing disability-based discrimination.
**Access to education**	Measures taken to ensure remote learning is fully accessible.
**Financial support**	Provision of financial support (e.g. cash transfers or benefits), to disabled people and their family members, if they had to stop working, and measures taken to ensure access to financial support, including automatic extension of disability benefits.
**Protection of people living in residential settings**	Measures taken to ensure people living in residential care are protected from infection.
**Reasonable accommodations for disabled people**	Adjustments to public health measures to accommodate the needs of disabled people, including flexibility in restrictions on movement in public spaces.
**Consideration of the needs of disabled people who face multiple exclusions**	Measures taken to protect disabled people who are in increased risk of social exclusion and poverty, such as women, children, homeless people and prisoners.
**Inclusion to decision making process**	Inclusion of disabled people and their representative organisations to advisory and decision-making bodies.

## Findings

We analysed 72 documents (Supplementary material 1). The findings illustrate the extent to which measures taken by four South American countries are disability-inclusive. Table 3 presents the government responses in each country. There was a wide range of policies implemented as a response to the COVID-19 pandemic but not all of these policies explicitly considered the needs of disabled people. Peru was the only country that voted legislation specifically protecting the rights of disabled people during the pandemic and ensuring their equal treatment in health, employment, education, social protection and other areas, with explicit reference to the CRPD. Several countries published recommendations for a disability-inclusive response to COVID-19, but these often either remained recommendations (especially in Brazil), without being translated into policy, or they put the responsibility on individual rather than state actors, asking people to protect themselves and others, without addressing disabled people’s needs. This is exemplified by policies in all four countries asking disabled people to shield, without always measures taken to ensure this is feasible. In Brazil and Chile, this was also evident for care home residents, who despite being particularly vulnerable to the risk of COVID-19 infection and death [27], were not protected by legislation or decisions from the central government, but only by recommendations.

**Table 3:** Disability inclusiveness of government responses to COVID-19

Area	Argentina	Brazil	Chile	Peru
**Relevant and accessible information**	• Information directly related to disabled people provided^16^. • Information line via video-call with sign-language interpretation, and through a dedicated online channel^16,17^. • Published recommendations specifically for disabled people^18^.	• Simultaneous sign language interpretation provided through specific software (VLIBRAS)^38^. • Information available in high contrast in all governmental webpages. • Published recommendations specifically for disabled people^32, 34.^ • Published recommendations specifically for indigenous people^24^.	• Video- calls, with sign language interpretation, available through a website^51^. • Information available in easy-read^49^. • Published recommendations specifically for disabled people^50^.	• All information, provided by any means, must be in accessible formats^58^.
**Access to healthcare**	• All services of the Programa Federal Incluir Salud are available online, including day centres and rehabilitation^6,10,12,19^.	• Telemedicine available for people with COVID-19 related symptoms^25^. • All general health appointments are suspended, but variations exist across states^25^.	• The GES programme (aimed at people diagnosed with one of currently 85 conditions with the objective of reducing inequalities in healthcare access) was suspended between March 30 and April 30^54^.	• Establishment of a community network to identify severely disabled people in the community, monitor their wellbeing, and offer support^64,65^. • Diagnostic services for suspected cases and treatment services are provided free of charge^63^. • Home visits to vulnerable people over 65 years of age^60^.
**Access to education**	• All services of the Programa Federal Incluir Salud are available online, including all levels of special education. If students do not have access to the internet, they are sent hard copies of educational material^10^. • Free access to online educational platforms (no data charges)^4^. • Reduction of educational support provision^6,22^. • Mainstream education: The national program ‘Seguimos educando’ does not make specific reference to disabled students^5^.	• National authorisation, exceptionally, allows the switch from face-to-face to digital classes in higher education. No specific reference to disabled students^26^. • In each state, measures to offer educational alternatives and provide internet access to students from public schools (primary and secondary education is the responsibility of states and municipalities). • Distribution of educational materials for disabled students^32^. • Distribution of food parcels to parents or guardians of state primary school students^27^.	• Extra financial support given to facilitate reasonable adjustments, like sign language interpretation. These are not available to students who have an open litigation case with the Servicio Nacional de Discapacidad^48^. • General measures do not specifically take into account the needs of disabled students^46,47^.	• Accessible education activities via the programme ‘Aprendo en casa’^60^. • Mainstream education: The national guidelines make explicit reference to the needs of disabled students and provide specific measures to promote inclusive education^62^.
**Employment and Financial support**	• Disabled people who receive a disability pension are entitled to an extra cash transfer of 3000 Argentine pesos (USD 44)^8^. • New disability registrations can happen via distance^7^. • Automatic renewal for 3 months (six in the Autonomous City of Buenos Aires) to all disability registrations that are about to expire^3,13^. • In order to safeguard jobs, an agreement can be signed between companies and corresponding unions, with a duration of 60 days starting 29/04. The payment of wages may not be less than 75% of the net wages. The State pays 50% of wages through the Work and Production Assistance Programme^23^.	• Suspension of “proof of life” (prova de vida) for beneficiaries of retirement and other social benefits, including disabled people^28^. • New disability registrations can happen via distance^98^. • Advance payment of R$200,00 (USD $40) for disabled people waiting for medical evaluation to receive financial benefits^29^. • Households with a disabled member are entitled to an extra financial benefit (usually it is ¼ of minimums salary, but it has increased to ½during the pandemic)^29^. • Emergency aid of two payments of R$600,00 (USD 120) each for freelancer workers or those informally employed^30^. • Temporary law allows salary reduction, with workload reduction, and contract suspension to keep jobs and income^30^.	• Disabled people who receive a disability pension are not entitled to the Covid-19 cash transfer of 50,000 Chilean pesos (USD 62), but disabled people who belong to vulnerable households are entitled to it^42,43^. • Family emergency income: for families of 2 people and above that are within the 60% poorest population. Amount depends on family characteristics, and will be given for 3 months, starting in May^42,43^. • Disability registrations continue but in order to certify disability, people need to go physically to the appropriate office^56^. • The Employment Protection Law allows workers to access the benefits and supplements of Unemployment Insurance, in case of contract suspension or working day reduction. It can apply from April 6th^,^ 2020 for up to six months^55^.	• Households with a disabled member are entitled to an extra cash transfer of 380 soles (USD111) per household^70^. • Paid leave for disabled people who cannot carry on working during the pandemic^58^. • In order to compensate for working time lost to quarantine, agreements can be made for the workers to take vacations, compensate for hours lost with accumulated overtime, accept reduced wages and working hours. If an agreement cannot be reached, the employer can unilaterally impose these measures^59^.
**Protection of people living in residential settings**	Recommendations for: • Prohibition of all visits^14^. • Suspension of all therapeutic and recreative outings^14^. • Obligatory self-isolation in their room for those residents who return to the facility^14^. • Suspected cases need to be referred to health system within 2 h of identifying them, and residents need to be moved to secondary care^14^.	• Recommendations for preventive actions in institutions for elderly people, including suggestions of visiting restrictions^37^.	Recommendations for: • Prohibition of all visits^52^. • Suspension of health appointments, unless they are necessary^52^. • Suspected cases need to be referred to health system and remain physically isolated^52^.	• Prohibition of all visits^68,69^.
**Reasonable accommodations for disabled people**	• Disabled people, who are registered as disabled, and their carers, are able to go for brief walks at a distance of up to 500 m from their houses up to three times a week, without needing a special permission^1,9^.	• Recommendations published by many organizations and universities, but no formal government policy.	• People with autism spectrum disorders and other mental health conditions, who are registered as disabled or have a doctor’s certificate, and their carers receive permission to be out for 2 h at a time, without restriction as to how many permissions they can receive^41^. • Carers of disabled people receive permission to go to work, even in areas that are under quarantine measures, if they work in care homes for disabled people^41^.	• Priority access to humanitarian supplies and all other resources provided by the State, at all levels of public administration (including water and food)^58,64^. • All measures directed to disabled people also include people who care for a family member^64^. • People with autism spectrum disorders, learning disabilities, and mental health conditions , and their carers, can be out of their house, but close to it, without needing a special permission, despite prohibition of movement^58,66^. • Disabled people can self-certify their disability status in order to receive associated benefits^58^. • Carers of disabled people receive permission to go to work^57^.
**Consideration of the needs of disabled people who face multiple exclusions**	• Assistance line for gender-based violence, with special reference to disabled women (but without communication accommodations)^11^. • Disabled prisoners are considered for early release^20^.	• Disabled prisoners are considered for early release (but new law being prepared at the time of writing, May 2020, will no longer allow this)^36^. • Information about women violence, including disabled people, and a special assistance line^31,35^. • Increased resources for the reporting of human rights violations, including against disabled people^33^.	• No information	• Disabled people at situations of risk receive priority attention by the Ministry of Women and Vulnerable Populations^64^.
**Inclusion to decision making process**	No information	• No information	• No information	• No information

Note 1: Exchange rates used as of May 26th, 2020: 1 ARS = 0.01 USD, 1 BRL = 0.19 USD, 1 CLP = 0.0012 USD, 1 PEN = 0.29 USD

Note 2: Superscript numbers indicate the number of the document from Supplementary material 1 where the information was found

Concerning health, Argentina and Peru took measures to protect disabled people’s continued access to healthcare, through enabling telehealth and/or ensuring COVID-19-related services were offered free of charge. Peru introduced measures to monitor the well-being of disabled people in the community, thus ensuring not only access to health, but also protection from violence. Other countries either did not take any measures to enable people’ access, or took measures that could have a detrimental impact on healthcare access. For example, the Explicit Guarantees in Health (GES) programme in Chile, which removes financial barriers of access to health for people diagnosed with certain chronic conditions [28], was suspended for one month, without a strategy to ensure continued access to healthcare. On a positive note, in Chile during the quarantine, people have been able to attend a doctor’s appointment at a health establishment using a special permit with a time limit of up to 12 h; in this case, they can be accompanied by one person. Measures, such as ensuring priority access for testing of disabled people and their carers, were not widely observed.

Regarding financial support measures, there was divergence between countries. We found examples of good practice, like the automatic renewal of disability registrations in Argentina and their inclusion in the extra cash transfers programme, and the possibility of self-certification in Peru, or remote registration in Brazil. In Chile, disabled people who received a disability pension were not entitled to the COVID-19 cash transfer that was meant to help the most vulnerable parts of the population. Furthermore, none of these four countries acknowledged the extra costs associated with disability, and how these might be further increased as a result of the pandemic (for example, due to the need to buy antiseptic products and face masks, or due to the inability to access usual ways of support).

Concerning education, several countries attempted to ensure continuity of education provisions. A good example of such practice was the law regarding educational provision in Peru, which made explicit provisions for disabled students. We also observed the existence of measures with opposing effects; Argentina, for example, ensured continuity of education provision through the use of online platforms (or hard copies of educational material where necessary), but at the same time significantly reduced the hours of educational support students are entitled to. Furthermore, while some countries had cash transfer programmes to help with adjustments, like equipment needs, others did not, affecting continued accessible educational provision.

## Discussion

Social inequalities and underfunded healthcare systems make South American countries vulnerable to the effects of the pandemic [29]. Disabled people are especially exposed to the effects of the pandemic, and to the ensuing socio-economic repercussions [15]. This study illustrates how the official government responses taken by four countries in the region do not fully address the needs of disabled people, thus further disadvantaging them. We have also seen positive steps that have been taken by these countries, in an attempt to protect the rights of disabled people during the COVID-19 pandemic. Additionally, in some instances, there is a discrepancy between central government policy and actual practice at the state or local levels (e.g. in Brazil), where measures implemented for the benefit of vulnerable groups, such as disabled people, may be contrary to the central government discourse.

Even where appropriate measures are available, they do not benefit all disabled people. In many countries in South America, disabled people need to be registered with a governmental agency, which certifies their status as disabled people, thus giving them access to an array of benefits. Such registration, however, is often incomplete and many disabled people are not officially registered, either because they are not eligible, the procedure is inaccessible, complicated (for example, requiring documents that people may not have), or for other reasons. Therefore, many people are excluded from several of the measures established by governments to protect disabled people. Already at increased risk to face multidimensional poverty [30], disabled people in South America might be inadequately protected against the risk of further impoverishment as a result of the economic consequences of COVID-19.

This study highlights how state mechanisms still consider disability as a personal matter. This is exemplified by measures in several countries, pointing to the need for disabled people and their families to protect themselves, to shield from the outside world, without always measures taken (e.g. cash transfers, continued access to social support, benefits, education, and health) to ensure their needs are met and their rights are protected. The different policies reported – while beneficial– do not constitute a collective matter of state responsibility. Disability becomes an individual responsibility; this *individualisation* exposes the most vulnerable parts of the population to the risk of poverty, and compromises their right to education, and lack of access to healthcare, by not providing reasonable adjustments. This fact was further underlined in a recent UK survey that found that 60% of disabled people are experiencing problems accessing food, medicine, and other necessities [31].

The barriers disabled people face when they seek to access healthcare are well-documented: long waiting times, discrimination, high costs, and transportation issues [32], even in countries in the region with universal health coverage [33]. Neoliberal reforms in several countries have disproportionately affected access to health for disabled people [34]. These issues are likely to become more acute due to the pandemic, with a higher probability that disabled people across the world, who are often precariously employed, becoming unemployed [3, 15, 35], and thus losing benefits such as insurance.

The intersectional nature of the disadvantage many disabled people face has not been adequately recognised and it has not informed policy. For example, while the intersections between disability and poverty, and disability and indigeneity are well-known [6], very few measures addressed this issue. Gender violence affects disabled women, but they might not be able to remove themselves from a dangerous situation, and assistance mechanisms may not be accessible to them. As illustrated by the few COVID-19-related welfare system measures implemented, social vulnerability is not at the top of the policy emergency agenda in South America. This is an indication of the historical presence of the neoliberal model in the region, according to which the role of the state should be minimal, thus leading to the formulation of poor policy responses for the protection of vulnerable groups, including disabled people, especially in the face of emergencies.

The CRPD was signed by 163 countries around the world, including all countries in South America; most of them (including the four countries in this study) have ratified it. Despite positive steps taken by many countries, it is a matter of concern to observe that several years after the ratification of the convention, and in the face of a pandemic, the rights of disabled people are not fully respected, and their needs are not fully considered by those state mechanisms responsible for the welfare of all citizens. Furthermore, and despite an explicit recommendation by the 2030 Agenda for Sustainable Development (equality target 17.18) for data disaggregated by disability, there is still no data available about the impact of COVID-19 on disabled people, including serious illness and death rates. To ensure that the measures taken by governments do not actively discriminate or put at an increased risk disabled people, it is important to disaggregate data and attend to the circumstances of those constructed as ‘vulnerable’.

The existence of official government response in the form of legislation is important for two main reasons: firstly, it affords public recognition for the parts of the population that are included therein, in the sense that they are named and thus become visible; secondly, it provides recognition and protection of the rights of disabled people, giving access to disabled people to ways to claim their rights (exemplified by the increasing judicialisation of healthcare in Brazil and Chile [36]). While there may be a gap between state discourse and actual practice, the existence of such a discourse provides disabled people with mechanisms to demand their rights.

## Strengths and limitations

The methods of data collection via official channels of information dissemination (for example, government gazettes) ensured the authenticity of all documents. This method of data collection also provided information on the provenance of all documents, ensuring a clear audit trail. In this study, we only looked at the national or federal government responses to the COVID-19 pandemic, based on legislation and other forms of state discourse. This meant that forms of disability exclusion (and inclusion) that were organised at the other levels of administration (e.g. by specific municipalities or states, in countries with a federal structure), or by the third sector, were not included. Furthermore, we did not look specifically at medical bias against disabled people and ableism in medical and scientific practice, neither to bioethical responses to disability and COVID-19. We only used documents that were available online. However, all four countries included in the study are legally obliged to publish online all legislation, official decrees, and other information that is considered as public.

## Recommendations

### Action needed

Most countries have published guides for disability inclusion in the COVID-19 response, but these often do not translate into practice. Action, in collaboration with disabled people, is needed. Disabled people and their representative organisations need to be involved in the decision-making process.

### Twin track approach

As Kuper and Heydt argue [32], a twin track approach is needed: disabled people need to be considered both in mainstream policy and in disability-specific policy, on issues such as communication and information sharing, education, health, employment, and social support.

### Build back better

Disabled people need to be included in the medium and long-term plans once the immediate crisis subsides. Several countries in Europe have already announced plans to allow coffee shops and other food and drink business to use up public space, such as pavements, without special permit; if this were to happen it could further disadvantage disabled people, who will find that urban landscapes become even more inaccessible. Recovery efforts need to be inclusive [37], so that disabled people are not further disadvantaged.

## Supplementary information

**Additional file 1: Supplementary material 1.** List of sources.

**Additional file 2: Supplementary material 2.** Recommendations for disability-inclusive response to COVID-19 by international organisations.

## Data Availability

The datasets analysed during the current study are available in the web addresses provided in Supplementary Material 1.

## Abbreviations

ARS: Argentine Peso
BRL: Brazilian Real
CLP: Chilean Peso
COVID-19: Coronavirus disease 19
CRPD: Convention on the Rights of Persons with Disabilities
ECLAC: Economic Commission for Latin America and the Caribbean
GDP: Gross Domestic Product
GES: Explicit Guarantees to Health [Garantías Explícitas en Salud]
PEN: Peruvian Sol
PPP: Purchasing Power Parity
USD: United States Dollar
WHO: World Health Organisation
