# Supplementary material for: Disability inclusiveness of government responses to COVID-19 in South America: a framework analysis study
Source: Int J Equity Health. 2020 Aug 3;19:131. doi: 10.1186/s12939-020-01244-x (PMC7396888; doi:10.1186/s12939-020-01244-x)
Supplement: Supplementary file 1 — Additional file 1: Supplementary material 1. List of sources. [file 12939_2020_1244_MOESM1_ESM.docx]

**Supplementary material 1: List of sources**

| **Country** | **#** | **Document** | **Source** |
| --- | --- | --- | --- |
| **Argentina** | | | |
|  | 1 | Resolution No 71, Article 6: Procedure for the authorization of exceptional sanitary and / or humanitarian and / or supply transfers [Procedimiento para la autorización de traslados excepcionales de carácter sanitario y/o humanitario y/o de abastecimiento] | Ministry of Transport  http://servicios.infoleg.gob.ar/infolegInternet/anexos/335000-339999/336380/norma.htm |
|  | 2 | Decree 297/2020. Preventive and obligatory isolation [Aislamiento social preventivo y obligatorio] | Ministry of Justice and Human Rights  https://www.boletinoficial.gob.ar/detalleAviso/primera/227042/20200320 |
|  | 3 | Disability certificate: Issuance and renewal [Certificado de discapacidad: otorgamiento y renovación] | Government of the Autonomous City of Buenos Aires  https://www.buenosaires.gob.ar/tramites/certificado-de-discapacidad |
|  | 1. 4 | 1. The Ministry of Education and Enacom agree free access to educational platforms from mobile phones [El Ministerio De Educación De La Nación y el Enacom acuerdan el acceso gratuito a las plataformas educativas desde los celulares] | Ministry of Education  https://www.argentina.gob.ar/noticias/el-ministerio-de-educacion-de-la-nacion-y-el-enacom-acuerdan-el-acceso-gratuito-las |
|  | 1. 5 | 1. Resolution 106/2020 | Ministry of Education  http://servicios.infoleg.gob.ar/infolegInternet/anexos/335000-339999/335490/norma.htm |
|  | 1. 6 | 1. Resolution 308/2020 | Superintendency of Health Services  https://www.argentina.gob.ar/normativa/nacional/resolución-308-2020-336194 |
|  | 7 | Resolution 106/2020 | National Agency of Disability https://www.boletinoficial.gob.ar/detalleAviso/primera/228513/20200501 |
|  | 8 | Resolution 93/2020 | National Agency of Disability https://www.boletinoficial.gob.ar/detalleAviso/primera/228238/20200424 |
|  | 9 | Resolution 77/2020 | National Agency of Disability https://www.boletinoficial.gob.ar/detalleAviso/primera/227719/20200413 |
|  | 10 | Resolution 69/2020 | National Agency of Disability https://www.boletinoficial.gob.ar/detalleAviso/primera/227370/20200402 |
|  | 11 | Help-line 144 | National Agency of Disability https://www.argentina.gob.ar/noticias/linea-144-atencion-mujeres |
|  | 12 | Resolution 60/2020 | National Agency of Disability https://www.boletinoficial.gob.ar/detalleAviso/primera/226928/20200318 |
|  | 13 | Resolution 63/2020 | National Agency of Disability https://www.boletinoficial.gob.ar/detalleAviso/primera/227055/20200320 |
|  | 14 | Guide of recommendations from the National Agency of Disability to places offering residential care to disabled people during the Covid-19 pandemic [Guia de recomendación de la agencia nacional de discapacidad para hogares y residencias que alberguen personas con discapacidad en el marco de la pandemia CONVID-19] | National Agency of Disability https://www.argentina.gob.ar/noticias/guia-de-recomendaciones-para-hogares-y-residencias-que-albergan-personas-con-discapacidad |
|  | 15 | Extra voucher for non-contributive pensions [Bono extraordinario para pensiones no contributivas y asignación universal] | National Agency of Disability https://www.argentina.gob.ar/noticias/bono-extraordinario-para-las-personas-con-discapacidad-que-cobran-pensiones-no |
|  | 16 | The National Agency of Disability provides you with information regarding coronavirus [Andis te acerca recomendaciones sobre coronavirus] | National Agency of Disability<https://www.argentina.gob.ar/andis/coronavirus-covid-19-0> |
|  | 17 | Video-call service for people with hearing impairment [Servicio de videollamada para personas sordas e hipoacúsicas] | National Agency of Disabilityhttps://www.argentina.gob.ar/noticias/servicio-de-videollamada-para-personas-sordas-e-hipoacusicas |
|  | 18 | Protocol of recommendations for caring and emotionally supporting disabled people [Protocolo de recomendaciones de asistencia y apoyo emocional para personas con discapacidad] | Ministry of Healthhttps://www.argentina.gob.ar/sites/default/files/covid-19-recomendaciones-asistencias-personas-discapacidad.pdf |
|  | 19 | Resolution 85/2020: Basic comprehensive care system for people with disabilities [Sistema de prestaciones básicas de atención integral a favor de las personas con discapacidad] | Boletin Oficial de la Republica Argentina [Official journal of the federal government of Argentina]  https://www.boletinoficial.gob.ar/detalleAviso/primera/227885/20200416 |
|  | 20 | Agreement 9/2020 | National Supreme Court of Justice  https://www.csjn.gov.ar/documentos/descargar/?ID=121993 |
|  | 21 | Decree 309/20: Health Emergency [Emergencia Sanitaria] | The Argentine Republic  https://www.boletinoficial.gob.ar/detalleAviso/primera/227114/20200324 |
|  | 22 | Inclusive Education [Educacion Inclusiva] | Civil Association for Equality and Justice  https://acij.org.ar/la-superintendencia-de-servicios-de-salud-redujo-la-cobertura-de-las-prestaciones-de-apoyo-a-la-educacion-de-los-ninos-y-ninas-con-discapacidad/ |
|  | 23 | Decree 329/2020 | The National Executive  https://www.argentina.gob.ar/normativa/nacional/decreto-329-2020-335976/texto |
| **Brazil** | | | |
|  | 24 | Epidimiological bulletin on coronavirus in indigenous populations [Boletim Epidemiológico do coronavirus em indígenas] | Ministry of Health  https://saudeindigena.saude.gov.br |
|  | 25 | Coronavirus | Heath Ministry:  <http://www.saude.gov.br/coronavirus> |
|  | 26 | Decree 343 | Diário Oficial da União [Official journal of the federal government of Brazil]  http://www.in.gov.br/en/web/dou/-/portaria-n-343-de-17-de-marco-de-2020-248564376 |
|  | 27 | Law 13.987 | Diário Oficial da União [Official journal of the federal government of Brazil]  http://www.in.gov.br/en/web/dou/-/lei-n-13.987-de-7-de-abril-de-2020-251562793 |
|  | 28 | Decree 373 | Diário Oficial da União [Official journal of the federal government of Brazil]  http://www.in.gov.br/en/web/dou/-/portaria-n-373-de-16-de-marco-de-2020-248328921 |
|  | 29 | Law 13.982 | Presidency of the Republic  http://www.planalto.gov.br/ccivil_03/_ato2019-2022/2020/lei/L13982.htm |
|  | 30 | Provisional Measure 936, April 1st, 2020 | Presidency of the Republic  http://www.planalto.gov.br/ccivil_03/_Ato2019-2022/2020/Mpv/mpv936.htm |
|  | 31 | Addressing domestic and family violence against women [Enfrentando a violência doméstica e familiar contra a mulher] | Ministry of Women, Family, and Human Rights  https://www.gov.br/mdh/pt-br/assuntos/noticias/2020-2/maio/cartilha-auxilia-mulheres-no-enfrentamento-a-violencia/Cartilhaenfrentamento_QRCODE1.pdf |
|  | 32 | Strategies for children with deficiencies and their families regarding home schooling [Estratégias para crianças com deficiências e suas famílias no acompanhamento escolar em casa] | Ministry of Women, Family, and Human Rights  https://sway.office.com/VLf4k28zYjefB3QD?ref=Link |
|  | 33 | Ministry receives 1,300 human rights violation reports during the novel coronavirus pandemic [Ministério recebe 1,3 mil denúncias de violações de direitos decorrentes da pandemia do novo coronavírus] | Ministry of Women, Family, and Human Rights  https://www.gov.br/mdh/pt-br/assuntos/noticias/2020-2/marco/ministerio-recebe-1-3-mil-denuncias-de-violacoes-de-direitos-decorrentes-da-pandemia-do-novo-coronavirus?_authenticator=a64c6642219d691acf1d3ab54905a4da7fcc1aec |
|  | 34 | Disabled people and people with rare diseases and Covid-19 [Pessoas com deficiência e com doenças raras e o Covid-19] | Ministry of Women, Family, and Human Rights  https://sway.office.com/tDuFxzFRhn1s8GGi?ref=Link |
|  | 35 | Line 100 opens an exclusive channel to receive complaints related to coronavirus [Disque 100 abre canal exclusivo para receber denúncias relacionadas ao coronavírus] | Ministry of Women, Family, and Human Rights  https://www.gov.br/mdh/pt-br/assuntos/noticias/2020-2/marco/disque-100-abre-canal-exclusivo-para-receber-denuncias-relacionadas-ao-coronavirus?_authenticator=617c55b7dfb348cf514d7fd04d1095f35adc8ee0 |
|  | 36 | Recommendation N.62/2020 | National Consul of Justice:  https://www.cnj.jus.br/wp-content/uploads/2020/03/62-Recomendação.pdf |
|  | 37 | Technical Note 5/2020 | Sanitary Vigilance National Agency  http://portal.anvisa.gov.br/documents/33852/271858/NOTA+TÉCNICA+Nº+05-2020+GVIMS-GGTES-ANVISA+-+ORIENTAÇÕES+PARA+A+PREVENÇÃO+E+O+CONTROLE+DE+INFECÇÕES+PELO+NOVO+CORONAVÍRUS+EM+INSTITUIÇÕES+DE+LONGA+PERMANÊNCIA+PARA+IDOSOS%28ILPI%29/8dcf5820-fe26-49dd-adf9-1cee4e6d3096 |
|  | 38 | VLIBRAS | Official homepage of the Federal Government:  <https://www.vlibras.gov.br> |
| **Chile** | | | |
|  | 39 | Sanitary controls: All about quarantines, zones,  and measures of isolation [Controles sanitarios: Todo sobre cuarentenas,  cordones y medidas de aislamiento] | The Government of Chile  https://cdn.digital.gob.cl/public_files/Campañas/Corona-Virus/documentos/Controles_Sanitarios_11042020.pdf |
|  | 40 | Total quarantine: Questions and answers [Cuarentena total: Preguntas y respuestas] | The Government of Chile  https://cdn.digital.gob.cl/public_files/Campañas/Corona-Virus/documentos/FAQ_Cuarentena_20200515.pdf |
|  | 41 | Guidance for movement permits [Instructivo para permisos de desplazamiento] | The Government of Chile  https://cdn.digital.gob.cl/public_files/Campañas/Corona-Virus/documentos/Instructivo_Cuarentena_15052020.pdf |
|  | 42 | Emergency economic plans due to coronavirus [Planes económicos de emergencia por coronavirus] | The Government of Chile https://cdn.digital.gob.cl/public_files/Campañas/Corona-Virus/documentos/Plan-Economico-de-emergencia14-05-20.pdf |
|  | 43 | Emergency economic plan: Questions and answers [Plan económico de emergencia: Preguntas y respuestas] | The Government of Chile  https://cdn.digital.gob.cl/public_files/Campañas/Corona-Virus/documentos/Descargas/FAQ-Plan-Economico-de-emergencia.pdf |
|  | 44 | Practical guide for emotional wellbeing [Guía práctica de bienestar emocional] | Ministry of Health  https://cdn.digital.gob.cl/public_files/Campañas/Corona-Virus/documentos/Guia-practica-cuarentena-en-tiempos-de-COVID19.pdf |
|  | 45 | Guidance for people in home isolation due to Covid-19 [Indicaciones para personas en aislamiento domiciliario por Covid-19] | Ministry of Health  https://www.minsal.cl/wp-content/uploads/2020/03/2020.03.13_INDICACIONES-EN-CUARENTENA.pdf |
|  | 46 | Protocol No 2: Coronavirus Covid 19 in educational establishments and prechools [Coronavirus Covid-19 en establecimientos educacionales y  jardines infantiles] | Ministry of Education  https://www.mineduc.cl/wp-content/uploads/sites/19/2020/03/circular_coronavirus.pdf |
|  | 47 | Action plan for higher education institutes [Plan de acción Mineduc para instituciones de educación superior] | Ministry of Education  https://educacionsuperior.mineduc.cl/wp-content/uploads/sites/49/2020/03/PLAN-DE-ACCION-EDUCACION-SUPERIOR-COVID-19-1.pdf |
|  | 48 | Resolution 673/20 | National Service of Disability  https://www.senadis.gob.cl/descarga/i/6060 |
|  | 49 | Constitutional state of exception of catastrophe: Coronavirus. Easy read [Estado de excepción constitucional  de catástrofe: Coronavirus. Lectura facil] | National Service of Disability  https://www.senadis.gob.cl/sala_prensa/d/noticias/8186/conoce-que-es-el-estado-de-excepcion-de-catastrofe-en-lectura-facil |
|  | 50 | Recommendations for disabled people regarding coronavirus [Recomendaciones para personas con discapacidad ante alerta de coronavirus] | National Service of Disability  https://www.senadis.gob.cl/region/antofagasta/d/portadas/8183/conoce-en-detalle-las-recomendaciones-para-personas-con-discapacidad-ante-el-coronavirus |
|  | 51 | Vi-Sor | National Service of Disability  https://www.vi-sor.cl/call/webcall |
|  | 52 | Protocol of recommendations for the prevention and care of Covid-19 in residential settings for disabled adults, phase 4 [Protocolo de recomendaciones para la prevención y atención del Covid-19 en residenciales para adultos con discapacidad, fase 4] | Ministry of Social Development and Family  https://cdn.digital.gob.cl/filer_public/69/21/6921ca49-4c6e-40bb-8f25-204d7b5e8747/protocolo_recomendaciones_a_residencias__para_pcd_coronavirus__1.pdf |
|  | 53 | Ministry of Health, Subsecretariat of Public Health, decrees health alert for the stipulated period and affords extraordinary powers, indicated for global public health emergency due to the outbreak of the novel coronavirus (2019- NCOV) [Ministerio de Salud, Subsecretaria de Salud Publica, decreta alerta sanitaria por el período que se señala y otorga facultades extraordinarias que indica por emergencia de salud pública de importancia internacional (ESPII) por brote del nuevo coronavirus (2019-NCOV)] | Diario Oficial de la Republica de Chile [Official journal of the government of Chile], No. 42.574. Ministry of Home Affairs and Public Security  https://www.minsal.cl/wp-content/uploads/2020/02/1724518_alerta_sanitaria_coronavirus.pdf |
|  | 54 | Ministry of Health, Susecretariat of Public Health, Suspends Guarantee of Opportunities of the Explicit Guarantees for Health for the Indicated Problems [Ministerio de Salud, Subsecretaria de Salud Publica, Suspende Garantía de Oportunidad de las Garantías Explícitas en Salud en los Problemas de Salud que Indica] | Diario Oficial de la Republica de Chile [Official journal of the government of Chile], No. 42.627. Ministry of Home Affairs and Public Security  https://www.diariooficial.interior.gob.cl/publicaciones/2020/04/08/42627/01/1749584.pdf |
|  | 55 | Employment protection law, COVID 19 [Ley de protección al empleo por COVID 19] | Library of the Nacional Congress of Chile  <https://www.bcn.cl/obtienearchivo?id=recursoslegales/10221.3/61435/4/Ficha_proteccion_empleo.pdf> |
|  | 56 | Registering in the National Disability Register | Civil Registry and Identification Service  https://www.chileatiende.gob.cl/fichas/3376-inscripcion-en-el-registro-nacional-de-la-discapacidad-rnd |
| **Peru** | | | |
|  | 57 | Supreme Decree No 044-2020-PCM | Presidency of the Council of Ministers. El Peruano [Official journal of the government of Peru], March 15, 2020  https://cdn.www.gob.pe/uploads/document/file/566448/DS044-PCM_1864948-2.pdf |
|  | 58 | Legislative Decree No 1468 | El Peruano [Official journal of the government of Peru], April 22, 2020  https://busquedas.elperuano.pe/normaslegales/decreto-legislativo-que-establece-disposiciones-de-prevencio-decreto-legislativo-n-1468-1865717-2/ |
|  | 59 | Urgent Legislative Decree 038-2020 | El Peruano [Official journal of the government of Peru], April 14, 2020  https://www.gob.pe/institucion/mtpe/normas-legales/473569-038-2020 |
|  | 60 | Ministerial Resolution 160-2020-MINEDU | El Peruano [Official journal of the government of Peru], April 1, 2020  https://cdn.www.gob.pe/uploads/document/file/574684/disponen-el-inicio-del-ano-escolar-a-traves-de-la-implementa-resolucion-ministerial-n-160-2020-minedu-1865282-1.pdf |
|  | 61 | Directorial Resolution 050-2020-MiDis/P65-De | El Peruano [Official journal of the government of Peru], March 15, 2020  https://cdn.www.gob.pe/uploads/document/file/575243/RD_050-2020-MIDISP65-DE.pdf |
|  | 61 | Viceministerial Resolution No 093-2020-MINEDU | Ministry of Education  https://www.gob.pe/institucion/minedu/normas-legales/535987-093-2020-minedu |
|  | 62 | Ministerial Resolution Mo 193-2020-MINSA | Ministry of Health  https://www.gob.pe/institucion/minsa/informes-publicaciones/473587-prevencion-diagnostico-y-tratamiento-de-personas-afectadas-por-covid-19-en-el-peru |
|  | 63 | Ministerial Resolution No 066-2020-MIDIS | Ministry of Development and Social Inclusion  https://cdn.www.gob.pe/uploads/document/file/581436/RM-066-MIDIS-2020.pdf |
|  | 64 | Viceministerial Resolution No 001-2020-MIDIS/VMPES | Ministry of Development and Social Inclusion  https://www.gob.pe/institucion/midis/normas-legales/484313-001-2020-midis-vmpes |
|  | 65 | Ministerial Resolution No 097-2020-PCM | Presidency of the Council of Ministers  https://www.gob.pe/institucion/pcm/normas-legales/481860-097-2020-pcm |
|  | 66 | Regulatory Compendium Covid-19 | Ministry of Women and Vulnerable Populations  <https://www.inabif.gob.pe/portalweb/img2/COMPENDIO.pdf> |
|  | 67 | Executive Resolution 0048 | National Integrated Programme for Family Wellbeing (INABIF)  http://www.inabif.gob.pe/portalweb/portal/portalweb/adicional/2020/rde_048_2020_2020-03-18-20-42-05.pdf |
|  | 68 | Executive Resolution 0052 | National Integrated Programme for Family Wellbeing (INABIF)  http://www.inabif.gob.pe/portalweb/portal/portalweb/adicional/2020/rde_052_2020_2020-03-31-22-09-11.pdf |
|  | 69 | Universal Family Voucher [Bono Familiar Universal] | Ministry of Work and Promoting Employment  https://www.bonoindependiente.pe |
| **All countries** | | | |
|  | 70 | COVID-19 Observatoryin Latin America and the Caribbean | Economic Commission for Latin America and the Caribbean, United Nations  https://www.cepal.org/en/topics/covid-19 |
|  | 71 | Persons with Disabilities and Coronavirus Disease (COVID-19) in Latin America and the Caribbean: Status and Guidelines | Economic Commission for Latin America and the Caribbean, United Nations  <https://www.cepal.org/en/publications/45492-persons-disabilities-and-coronavirus-disease-covid-19-latin-america-and-caribbean> |
|  | 72 | Covid-19 and the Rights of Persons with Disabilities: Guidance. | United Nations Office of the High Commissioner for Human Rights  <https://www.ohchr.org/Documents/Issues/Disability/COVID-19_and_The_Rights_of_Persons_with_Disabilities.pdf> |
