# Supplementary material for: Disability inclusiveness of government responses to COVID-19 in South America: a framework analysis study
Source: Int J Equity Health. 2020 Aug 3;19:131. doi: 10.1186/s12939-020-01244-x (PMC7396888; doi:10.1186/s12939-020-01244-x)
Supplement: Supplementary file 2 — Additional file 2: Supplementary material 2. Recommendations for disability-inclusive response to COVID-19 by international organisations. [file 12939_2020_1244_MOESM2_ESM.docx]

**Supplementary material 2: Recommendations for disability-inclusive response to COVID-19 by international organisations**

| **Organisation** | **Recommendations** |
| --- | --- |
| Economic Commission for Latin America and the Caribbean [3] | - Respect the fundamental and inalienable rights of all persons in the context of the crisis and in the recovery period, regardless of their disability status, and address in particular subgroups of the population that may be particularly disadvantaged, such as women, children and adolescents with disabilities. - Ensure that all information related to the crisis is accessible. - Ensure accessibility at testing sites for COVID-19 diagnosis and treatment. - Strengthen non-contributory social protection entitlements for persons with disabilities and their families. - Ensure that measures taken to address the crisis and during the recovery period incorporate the disability perspective, including measures relating to health, employment protection and educational continuity. - Create or expand spaces for participation and consultation with organizations of persons with disabilities. - Ensure the continuity of work and education and the provision of rehabilitation services for persons with disabilities. - Provide psychosocial support to persons with disabilities and their families and strengthen support networks. - Adopt flexibility in restrictions on movement in public spaces. - Improve the statistical information collected during and after the crisis. |
| International Labour Organization [24] | - Ensure that all information is inclusive and accessible. - Coordinate with organizations of persons with disabilities and other relevant stakeholders. - Remove any financial barriers to access health care. - Minimize risks of contamination of persons with disabilities across social protection delivery mechanisms. - Extend identification and registration of persons with disabilities. - Carry out needs assessment of persons with disabilities. - Increase the level of disability benefits, providing extra payments and/or advancing payments. - Extend cash transfers to all persons with disabilities officially registered that may not be eligible under regular circumstances. - Automatically extend any soon-to-expire disability related entitlements. - Provide financial assistance for persons who stop working to support or to prevent contamination of their family member(s) with disabilities. - In kind assistance and support services. - Ensure development and continuity in access to quality care and support. - Delivery of essential items to persons with high support needs. - Create helpline and other support platforms. - Ensure that economic recovery programs are inclusive of persons with disabilities and their families. |
| Secretaría General Iberoamericana [25] | - Provision of information in accessible formats. - Reasonable adjustments to ensure access to healthcare services, including for people who are self-isolating. - Measures to ensure disabled workers and disabled students are not disadvantaged. - Guarantee essential services are accessible, including transportation, food supply, cleaning, and disinfection. - Establish services specifically to address needs arising due to lockdown measures, which might disproportionately affect disabled people, with special attention at disabled girls and women, and to population of disabled people who are disadvantaged, including displaced people, migrants, refugees, indigenous people, rural populations, and conflict victims. |
| United Nations Office of the High Commissioner for Human Rights [4] | *Rights*   - Prohibit the denial of treatment on the basis of disability. - Priority testing of persons with disabilities presenting symptoms. - Research on the impact of COVID-19 on the health of persons with disabilities. - Identify and remove barriers to treatment. - Ensure the continued supply and access to medicines for persons with disabilities during the pandemic. - Training and awareness-raising of health workers to prevent disability-based discrimination. - Consult with and actively involve persons with disabilities and their representative organisations.   *Institutions*   - Discharge and release persons with disabilities from institutions. - Prioritise testing and preventive measures within institutions. - Increase temporarily the resources of institutions.   *Community*   - Ensure that information on COVID-19 related measures is accessible. - Promote and coordinate the development of community support networks. - Reasonable accommodations to persons with disabilities.   *Income*   - Provide financial aid for persons with disabilities with and without any income. - Extend automatically any soon-to-expire disability related entitlements. - Financial assistance programmes for persons who stop working to support or to prevent contamination of their family member(s) with disabilities. - Provide financial support to employers of persons with disabilities to provide equipment required for teleworking. - Ensure that food provision schemes include persons with disabilities.   *Education*   - Provide clear guidance to education and school authorities. - Ensure access to Internet for remote learning and ensure that all educational material are accessible. - Establish close coordination with parents and caregivers.   *Violence*   - Ensure that reporting mechanisms and other forms of assistance include persons with disabilities. - Carry out monitoring of the situation of persons with disabilities. - Raise awareness and provide training about the risk of violence faced by persons with disabilities.   *Prisoners, homeless*   - Reduce the prison population by releasing at-risk groups of prisoners, including persons with disabilities. - Implement preventive measures within prisons. - Ensure that homeless persons with disabilities are treated with dignity and respect and services are disability-inclusive. |
| World Health Organisation [26] | *Guidelines for governments*   - Ensure public health information and communication is accessible. - Undertake targeted measures for people with disability and their support networks. - Undertake targeted measures for disability service providers in the community. - Increase attention given to people with disability living in high-risk situations. - Ensure that emergency measures include the needs of people with disability. |
